# *Hox* genes pattern the anterior-posterior axis of the juvenile but not the larva in a maximally indirect developing invertebrate, *Micrura alaskensis* (Nemertea)

**DOI:** 10.1186/s12915-015-0133-5

**Published:** 2015-04-11

**Authors:** Laurel S Hiebert, Svetlana A Maslakova

**Affiliations:** Oregon Institute of Marine Biology, University of Oregon, Charleston, OR USA

**Keywords:** Biphasic life cycle, *Hox*, Indirect development, Larval evolution, Nemertea, Pilidium

## Abstract

**Background:**

The pilidium larva is a novel body plan that arose within a single clade in the phylum Nemertea - the Pilidiophora. While the sister clade of the Pilidiophora and the basal nemerteans develop directly, pilidiophorans have a long-lived planktotrophic larva with a body plan distinctly different from that of the juvenile. Uniquely, the pilidiophoran juvenile develops inside the larva from several discrete rudiments. The orientation of the juvenile with respect to the larval body varies within the Pilidiophora, which suggests that the larval and juvenile anteroposterior (AP) axes are patterned differently. In order to gain insight into the evolutionary origins of the pilidium larva and the mechanisms underlying this implied axial uncoupling, we examined the expression of the *Hox* genes during development of the pilidiophoran *Micrura alaskensis*.

**Results:**

We identified sequences of nine *Hox* genes and the *ParaHox* gene *caudal* through a combination of transcriptome analysis and molecular cloning, and determined their expression pattern during development using *in situ* hybridization in whole-mounted larvae. We found that *Hox* genes are first expressed long after the pilidium is fully formed and functional. The *Hox* genes are expressed in apparently overlapping domains along the AP axis of the developing juvenile in a subset of the rudiments that give rise to the juvenile trunk. *Hox* genes are not expressed in the larval body at any stage of development.

**Conclusions:**

While the *Hox* genes pattern the juvenile pilidiophoran, the pilidial body, which appears to be an evolutionary novelty, must be patterned by some mechanism other than the *Hox* genes. Although the pilidiophoran juvenile develops from separate rudiments with no obvious relationship to the embryonic formation of the larva, the *Hox* genes appear to exhibit canonical expression along the juvenile AP axis. This suggests that the *Hox* patterning system can maintain conserved function even when widely decoupled from early polarity established in the egg.

**Electronic supplementary material:**

The online version of this article (doi:10.1186/s12915-015-0133-5) contains supplementary material, which is available to authorized users.

## Background

The life history of many marine invertebrates is strikingly biphasic. In the most extreme cases, often termed maximally indirect development, adults and larvae differ so dramatically that they were originally described as different animals. Such development is exemplified by the pilidiophoran worms of the phylum Nemertea. During embryogenesis, a pilidium larva forms, complete with a domed episphere, apical sensory organ, blind stomach, and ciliated band for feeding and swimming. Days to weeks after the larval body plan is established, a juvenile begins to develop from distinct rudiments, called imaginal discs, that eventually fuse around the larval stomach. Once complete, the juvenile escapes the larval body and devours the larval tissues in a catastrophic metamorphosis.

**Figure 1:**
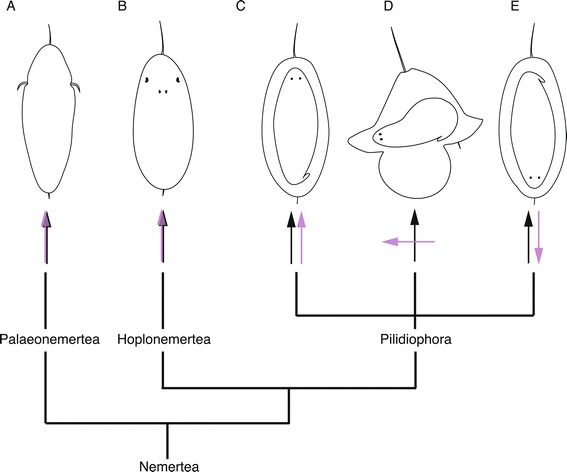
Distribution of larval types and larval-juvenile axial orientations among major Nemertean clades. Phylogeny (bottom) shows relationships among three major nemertean clades. Above: orientation of anteroposterior (AP) axis of larva (black arrows) and juvenile (purple arrows) of **(A)** a typical palaeonemertean larva, **(B)** a typical hoplonemertean larva, and **(C)** the larva of pilidiophoran *Micrura rubramaculosa* [62], in which the larval and juvenile AP axes coincide; **(D)** a typical pilidium, in which larval and juvenile axes are roughly perpendicular; and **(E)** the larva of *M. akkeshiensis*, in which larval and juvenile AP axes oppose one another.

The maximally indirect development of the pilidiophorans appears to be derived from more-or-less direct development [1-3]. The sister clade to the Pilidiophora (the Hoplonemertea) and the basal Palaeonemertea both have rather direct modes of development (Figure 1). Both have ciliated larvae that gradually grow into juvenile worms rather than passing through a morphologically distinct larval stage. Thus, the pilidium could be viewed as an insertion of a novel body plan into the life cycle of a more directly developing nemertean.

If the pilidium is a true novelty, one might expect different mechanisms patterning the larval versus juvenile body. Diversity of the orientation of the juvenile with respect to the larva within the Pilidiophora provides indirect evidence of such mechanistic decoupling. While all pilidiophorans build worm-shaped juveniles inside larval bodies, the anteroposterior (AP) axes of the larva and the juvenile appear to be decoupled. In a typical pilidium, the juvenile AP axis is roughly perpendicular to the larval AP axis (Figure 1D). In some pilidia, however, the AP axis of the juvenile coincides with that of the larva, as in the sock-like larvae called pilidium incurvatum [4] and pilidium recurvatum of *Riserius* sp. [4-7], the non-feeding bullet-shaped larvae of *Micrura rubramaculosa* and *M. verrilli* [8], and the trochophore-like pilidium nielseni [9,10] (Figure 1C). However, in the lecithotrophic larvae of *M. akkenshiensi*s [11] and several other species, the AP axis of the larva and juvenile oppose one another [11] (Figure 1E). This diversity suggests that pilidiophoran evolution may have been accompanied by the dissociation of axial patterning mechanisms between life history stages.

In order to understand the mechanics of pilidiophoran development and the origin of a novel larval body plan, we examined the expression of the *Hox* genes during development of a pilidiophoran nemertean *M. alaskensis. Hox* genes are highly conserved patterning genes that are expressed in and determine the identity of domains along the AP axis of many animals [12-14]. We also looked at expression of one of the three *ParaHox* genes, *caudal* (*Cdx*), which is a posterior marker in other animals [15].

Very little is known about patterning mechanisms in nemerteans [16-18]. Here, we report for the first time on the patterns of expression of *Hox* genes in this phylum, specifically during the development of the pilidium larva, and discuss the relevance of these findings to the origins of the pilidium larva.

## Results

### Development of *M. alaskensis*

**Figure 2:**
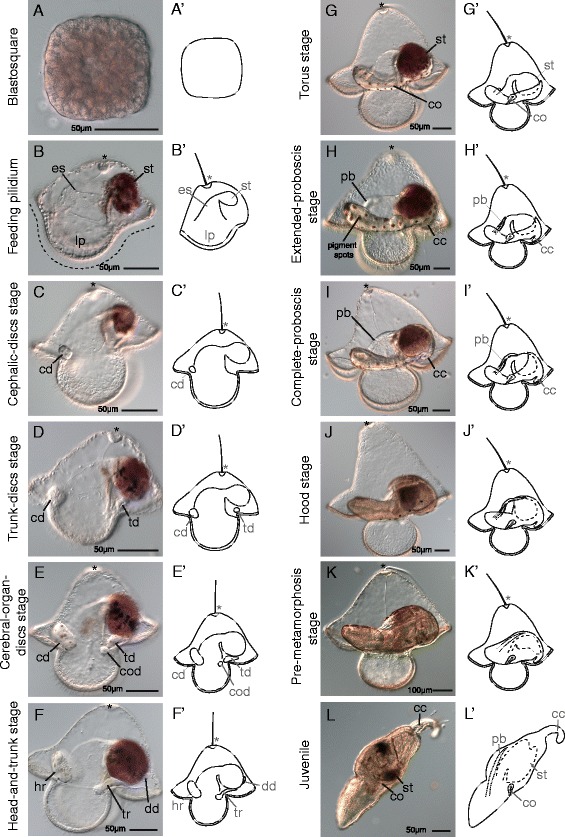
Development of *Micrura alaskensis*. Differential interference contrast images. Lateral views except in (A). Apical organ (indicated by asterisk) orientated up, juvenile anterior to the left. **(A)** Blastosquare stage. Polar view (animal or vegetal). **(B)** Feeding pilidium stage larva. Esophagus (es), leads to blind stomach (st), which is dark red due to algal food. Ciliated band, indicated with dashed line, spans lobes and lappets (lp). **(C)** Cephalic-discs-stage larva. One of the paired cephalic discs (cd) labeled. **(D)** Trunk-discs-stage larva. One of the paired trunk discs (td) labeled. **(E)** Cerebral-organ-discs-stage larva. One of the paired cerebral-organ discs (cod) labeled. **(F)** Head-and-trunk-stage larva. Cephalic discs have merged with each other to become the head rudiment (hr). Cerebral-organ discs have merged with the trunk discs to become the trunk rudiment (tr). Dorsal disc (dd) is present. **(G)** Torus-stage larva. All discs have merged together to form toroid of juvenile tissue around larval stomach. Cerebral organ (co) is labeled. **(H)** Extended-proboscis-stage larva. Proboscis (pb) has begun to grow over the esophagus towards the stomach. Caudal cirrus (cc) is evident. Membrane housing juvenile (the amnion) is decorated with red-brown pigment spots in a polka-dot pattern. Amniotic pigment spots develop in earlier stages, but are quite clear in this image. **(I)** Complete-proboscis-stage larva. Proboscis has grown to meet stomach. **(J)** Hood-stage larva. Dorsal tissues of juvenile overgrow proboscis. **(K)** Pre-metamorphosis-stage larva. Juvenile is complete inside larva. Metamorphosis (not shown) is rapid and radical; juvenile breaks out of larval body and devours all larval tissues in a minute or less. **(L)** Juvenile after metamorphosis. Larval structures, including amniotic pigment, are inside juvenile stomach.

The development and a staging scheme for *M. alaskensis* is documented in detail by Maslakova [19], and is briefly summarized here. Fertilized zygotes undergo spiral cleavage and pass through a square-shaped blastula (blastosquare) stage (Figure 2A). By day 1, they have developed into swimming gastrulae with an apical tuft. By day 2, a ciliated band spanning two lateral lobes becomes evident. At about 3 days, the larvae begin to feed on microalgae (Figure 2B). Food particles pass through the funnel-shaped esophagus, which is equipped with two ciliated ridges, and enter the blind gut. As the larva grows, the lappets grow larger and two additional elaborations of the ciliated band appear, called the lobes (anterior and posterior, in reference to the juvenile AP axis). The first rudiments of the juvenile, the cephalic imaginal discs, appear as invaginations of the larval episphere as early as 5 to 7 days, and give rise to the juvenile head. We refer to this stage as the ‘cephalic-discs stage’ (Figure 2C). The timing of development, especially the emergence of the juvenile rudiments, is highly variable, and depends on the water temperature, feeding regime, and, possibly, other factors. As the cephalic discs are growing, a pair of trunk discs invaginates from the hyposphere near the larval stomach. These discs give rise to the majority of the trunk of the juvenile. We refer to this stage as the ‘trunk-discs stage’ (Figure 2D). The third and final pair of imaginal discs, called the cerebral-organ discs, arise as early as 2 weeks, as invaginations of the inner epidermis of the larval lappets at the lower end of the esophageal ciliated ridges. These discs are located anterior to the trunk discs (along the AP axis of the future juvenile), and give rise to the cerebral organs. Thus, this stage is called ‘cerebral-organ-discs stage’ (Figure 2E). At about the same time, an unpaired proboscis rudiment appears as a small cluster of cells near the larval epidermis in-between the two cephalic discs. The proboscis rudiment later fuses with the cephalic discs to form the head rudiment, while the trunk and cerebral organ discs fuse with each other to form the trunk rudiments. An unpaired dorsal disc arises dorsal to the trunk rudiments between the stomach and the larval epidermis, at about the same time as the proboscis rudiment. This characterizes the ‘head-and-trunk stage’ (Figure 2F). As early as 4 weeks after fertilization, the dorsal disc fuses with the trunk rudiments and, subsequently, the head rudiment fuses with the trunk rudiment to form a toroid of juvenile tissue around the stomach. We refer to this stage as the ‘torus stage’ (Figure 2G). The juvenile proboscis continues to grow, first extending beyond the margin of the juvenile head (‘extended-proboscis stage’, Figure 2H), then reaching the dorsal margin of the juvenile trunk epidermis (‘complete-proboscis stage’, Figure 2I). The juvenile tissues grow over the stomach and esophagus (before fusing, ‘hood stage’, Figure 2J), eventually enclosing the gut (after fusing, ‘pre-metamorphosis stage’, Figure 2K). As early as 35 days, the juvenile ruptures the larval enclosure and engulfs the larval tissues in a radical metamorphosis.

### *M. alaskensis Hox* gene sequences

Nine contigs containing *Hox* gene sequences were recovered from the *M. alaskensis* developmental transcriptome. Full coding sequences were isolated from seven of the nine genes. Characteristic residues and motifs were found in all nine sequences and permitted assignment to known paralog groups [20-23]. See Additional file 1 for alignment of *M. alaskensis Hox* genes with those of other bilaterians. Bayesian phylogenetic analysis supports assignment to paralog groups (PG) based on the presence of characteristic residues (Additional file 2). *M. alaskensis* has representatives from PG1 (*Labial/Lab*), PG2 (*Proboscipedia/Pb*), PG3 (*Hox3*), PG4 (*Deformed/Dfd*), PG5 (*Sex combs reduced/Scr*), PG6 (*Lox5*), PG7 (*Antennapedia/Antp*), PG8 (*Lox4*), and PG9-PG13 (*Post2*). *Cdx* and *Six3/6* were also isolated and identified via phylogenetic analysis (see Additional file 2 for *Cdx*, data for *Six3/6* not shown). Additional file 3 lists details of each gene, including paralog group, length of predicted open reading frame, and GenBank accession number.

Six *Hox* genes and the *Cdx* gene have been previously cloned from a single nemertean species, the pilidiophoran *Ramphogordius (= Lineus) sanguineus* [17]; however, their expression had not been characterized. *M. alaskensis* genes *MaLab*, *MaHox3*, *MaDfd*, *MaLox5*, *MaAntp*, and *MaPost2* isolated by us are orthologous to *R. sanguineus* genes *LsHox1*, *LsHox3*, *LsHox4*, *LsHox6*, *LsHox7*, and *LsHox9,* respectively.

### Gene expression in the development of *M. alaskensis*

No *Hox* gene expression was detected at any stage of development in the larval body, cephalic discs, cerebral organ discs, or the proboscis rudiment. We are confident that the *in situ* hybridization protocol works on pilidial structures and early stages because we have expression data for other genes at early stages (for example, *MaSix3/6*, which is expressed at blastosquare and in the pilidial body during feeding-pilidium stage, see Additional file 4). *Hox* genes were expressed at various later developmental stages in different patterns in the trunk discs and the dorsal disc, and, as a rule, expression became more prominent, and occupied larger domains in more advanced developmental stages.

#### *Labial* (*MaLab*)

**Figure 3:**
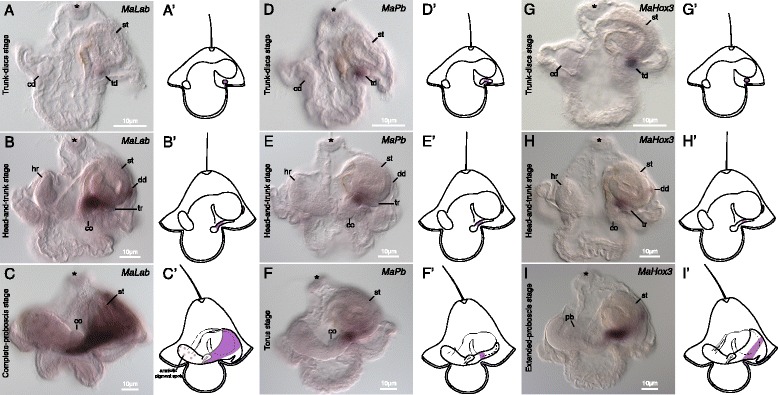
Expression of *MaLab, MaPb,* and *MaHox3* in *M. alaskensis* larval development. All images and diagrams show lateral views with larval anterior up and future juvenile anterior to the left. Asterisk marks apical organ. **(A)** *MaLab* is expressed in the trunk discs (td) at trunk-discs stage. The cephalic disc (cd) and stomach (st) are marked for reference. **(B)** In head-and-trunk-stage larvae, *MaLab* is expressed in portion of the trunk rudiment (tr) and dorsal disc (dd). The head rudiment (hr) is marked for reference. **(C)** In complete-proboscis-stage larvae *MaLab* is expressed in a large portion of the juvenile posterior, from just up against the cerebral organ (co). **(D)** *MaPb* is expressed initially in trunk discs (td) at trunk-discs stage. **(E)** In head-and-trunk-stage larvae, *MaPb* is expressed in a central domain of the trunk rudiment (tr). **(F)** In torus-stage larvae, *MaPb* is expressed in a small central domain of the juvenile posterior. **(G)** The onset of *MaHox3* expression occurs at the trunk-discs stage in the trunk discs (td). **(H)** In head-and-trunk-stage larvae, *MaHox3* is expressed in a central domain of the trunk rudiment (tr) and in a portion of the dorsal disc (dd). *MaHox3* is not expressed in the larval body or head rudiment (hr). **(I)** In extended-proboscis-stage larvae, *MaHox3* is expressed in a narrow stripe of the juvenile posterior. Proboscis rudiment (pb) marked in image. Anterior spots are amniotic pigment spots (see Figure 2) that happen to be in focus in this image. **(A’-I’)** diagrammatically illustrate expression patterns in the three respective developmental stages.

No expression was observed in gastrulae or young pilidia (data not shown). Weak expression of *MaLab* is first noticeable in the trunk discs as soon as they emerge (Figure 3A). At the head-and-trunk stage, expression becomes more prominent and occupies a broader domain in each trunk disc, and is also detectable in the dorsal disc (Figure 3B). In complete-proboscis-stage larvae, expression extends to the anterior margin of the trunk disc, bordering the cerebral organ, and posteriorly throughout most of the juvenile trunk, except the very posterior (Figure 3C).

#### *Proboscipedia* (*MaPb*)

Similar to *MaLab*, expression of *MaPb* is first evident in the trunk discs as soon as they emerge (Figure 3D). By head-and-trunk stage, *MaPb* is expressed clearly in the middle portion of the trunk rudiments, but not in the dorsal disc (Figure 3E). About a fourth of each trunk rudiment is stained, forming a stripe about midway from the anterior portion of the trunk discs to the posterior tip of the trunk (Figure 3E). By torus stage, the expression of *MaPb* remains localized to a fairly narrow region of the trunk about midway between the cerebral organ and the posterior end (Figure 3F).

#### *Hox3* (*MaHox3*)

As is the case with *MaLab* and *MaPb*, no expression of *MaHox3* is detectable until the trunk-discs stage, and expression is first evident in the trunk discs (Figure 3G). At the head-and-trunk stage, the expression domain occupies the anterior portion of the trunk discs (Figure 3H). The size of the expression domain is similar to *MaPb*. Unlike *MaPb*, expression of *MaHox3* at this stage is also detectable in the dorsal disc (Figure 3H). At extended-proboscis stage, the expression in the dorsal disc and the trunk discs forms a continuous band encircling the juvenile trunk about half way between the cerebral organs and the posterior end (Figure 3I).

#### *Deformed* (*MaDfd*)

**Figure 4:**
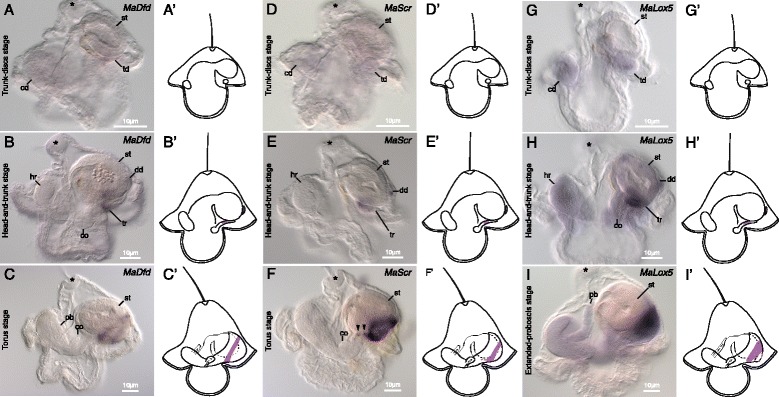
Expression of *MaDfd, MaScr,* and *MaLox5* in *M. alaskensis* larval development. All images and diagrams show lateral views with larval anterior up and future juvenile anterior to the left. Asterisk marks apical organ. **(A)** *MaDfd* expression is not evident during the trunk-discs stage anywhere in the larval or juvenile body, including the trunk discs (td), the cephalic disc (cd), and stomach (st). **(B)** *MaDfd* is first clearly expressed during the head-and-trunk stage in small domains of each trunk rudiment (tr) and in a portion of the dorsal disc (dd). The head rudiment (hr) is indicated for reference. **(C)** In torus-stage larvae, *MaDfd* is expressed in a very narrow ring of the juvenile posterior. Proboscis rudiment (pb) marked for reference. **(D)** *MaScr* expression is not evident during the trunk-discs stage anywhere in the larval or juvenile body. **(E)** The onset of *MaScr* expression occurs during the head-and-trunk stage in domains of each trunk rudiment (tr) and in a portion of the dorsal disc (dd). **(F)** In torus-stage larvae, *MaScr* is expressed in a ring of the juvenile posterior with an additional few small spots of expression laterally towards the anterior of the juvenile (arrowheads). **(G)** *MaLox5* expression is not evident during the trunk-discs stage anywhere in the larval or juvenile body. Faint background staining is observed in both pairs of discs. **(H)** The onset of strong *MaLox5* expression occurs during the head-and-trunk stage in domains of the each trunk rudiment (tr) and in a portion of the dorsal disc (dd). Faint background staining persists in all juvenile rudiments, including head rudiment (hr) as well as portions of the stomach. **(I)** *MaLox5* is expressed in a patch near the posterior end of the juvenile in extended-proboscis-stage larvae. **(A’-I’)** diagrammatically illustrate expression patterns in the three respective developmental stages.

*MaDfd* is not expressed at early stages, including the trunk-discs stage (Figure 4A). By the head-and-trunk stage, *MaDfd* is expressed in a very small patch in trunk rudiments, similar in location to *MaHox3*, but slightly more posterior, as well as in the anterior portion of the dorsal disc (Figure 4B). By torus stage, *MaDfd* is expressed in a very thin band around the juvenile trunk (Figure 4C).

#### *Sex combs reduced* (*MaScr*)

Similar to *MaDfd*, *MaScr* is not apparently expressed until the head-and-trunk stage (Figure 4E). At the head-and-trunk stage, *MaScr* is expressed in a region of both trunk rudiments and the posterior region of the dorsal disc (Figure 4F). The expression domain in the trunk rudiment is slightly wider than that of *MaPb*, *MaHox3*, and *MaDfd.* During the torus stage, *MaScr* is expressed in a thin belt around the juvenile trunk located somewhat more posterior to the domains of *MaPb*, *MaHox3*, and *MaDfd* (compare Figure 4F with Figures 3F,I and 4C). A few small localized spots of expression occur laterally on either side of the juvenile trunk just anterior to the main expression domain (Figure 4F).

#### *Lox5* (*MaLox5*)

The faint staining observed at early stages throughout the juvenile rudiments (see Figure 4G) likely represents either very weak expression or possibly just background staining (Figure 4G). Clear *MaLox5* expression is first detected at the head-and-trunk stage in the trunk rudiments and dorsal disc (Figure 4H). Within the trunk rudiments, the expression domain of *MaLox5* appears to be slightly broader than that of *MaScr*. In extended-proboscis-stage larvae, the expression domain appears as a broad band encircling the juvenile trunk near the posterior end (Figure 4I).

#### *Antennapedia* (*MaAntp*)

**Figure 5:**
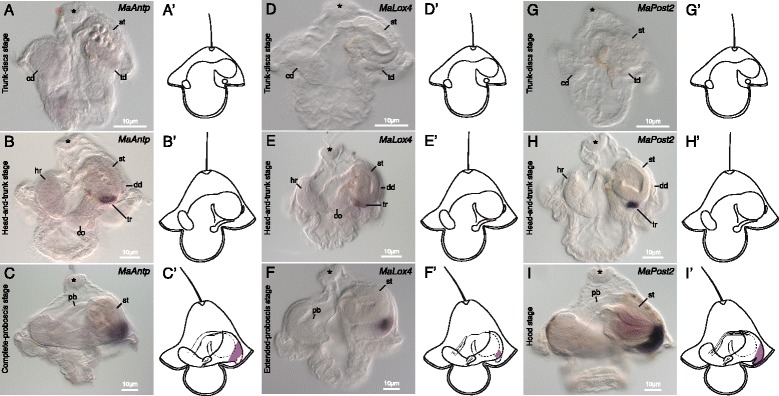
Expression of *MaAntp, MaLox4,* and *MaPost2* in *M. alaskensis* larval development. All images and diagrams show lateral views with larval anterior up and future juvenile anterior to the left. Asterisk marks apical organ. **(A)** *MaAntp* expression is not evident during the cerebral-organ-discs stage anywhere in the larval or juvenile body, including the trunk discs (td), the cephalic disc (cd), cerebral organ discs (cod), or stomach (st). **(B)** Initial *MaAntp* expression occurs during the head-and-trunk stage in domains of each trunk rudiment (tr) and in a portion of the dorsal disc (dd). The head rudiment (hr) is indicated for reference. **(C)** *MaAntp* is expressed in a patch near the posterior end of the juvenile in complete-proboscis-stage larvae. Proboscis rudiment (pb) marked for reference. **(D)** *MaLox4* is not expressed in trunk-discs-stage larvae. **(E)** *MaLox4* expression first occurs during the head-and-trunk stage in domains of each trunk rudiment (tr), with no evident expression in the dorsal disc (dd). **(F)** *MaLox4* is expressed in a small spot near the posterior end of the juvenile in extended-proboscis-stage larvae. **(G)** *MaPost2* is not expressed in trunk-discs-stage larvae. **(H)** *MaPost2* expression first occurs during the head-and-trunk stage in posterior domains of each trunk rudiment (tr), with no evident expression in the dorsal disc (dd). **(I)** *MaPost2* is expressed in the juvenile caudal cirrus in hood-stage larvae. **(A’-I’)** diagrammatically illustrate expression patterns in the three respective developmental stages.

Expression of *MaAntp* is first detectable at the head-and-trunk stage (Figures 5A,B). At this stage, *MaAntp* is expressed in posterior domains of the trunk rudiments and the posterior part of the dorsal disc (Figure 5B). By complete-proboscis stage, expression is confined to a fairly broad band encircling the juvenile trunk near the posterior end (Figure 5C).

#### *Lox4* (*MaLox4*)

Faint expression of *MaLox4* is first evident at the head-and-trunk stage in the trunk rudiments (Figure 5E). By the extended-proboscis stage, expression of *MaLox4* appears as a patch near the posterior of the juvenile (Figure 5F).

#### *Post2* (*MaPost2*)

*MaPost2* is not expressed in early stages (Figure 5G), and is first detectable at the head-and-trunk stage at the very posterior of the trunk rudiments (Figure 5H). No expression was observed in the dorsal disc. By the hood stage, *MaPost2* is expressed in a broad domain at the very posterior of the juvenile trunk including the juvenile caudal cirrus (Figure 5I).

#### *Caudal* (*MaCdx*)

**Figure 6:**
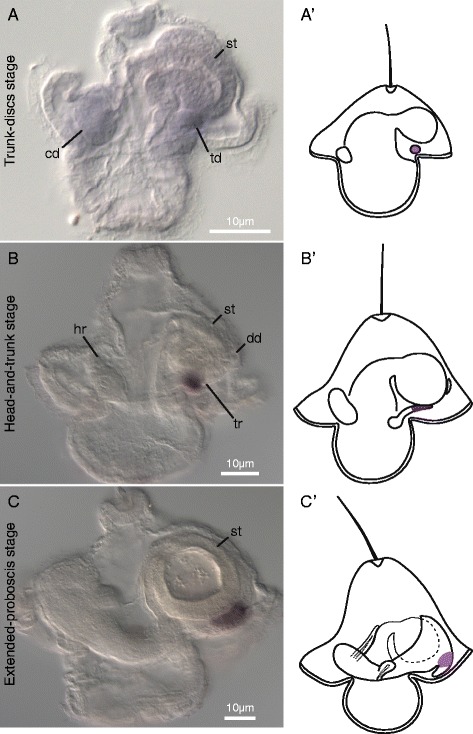
*MaCdx* expression in *M. alaskensis* larval development. All images and diagrams show lateral views with larval anterior up and future juvenile anterior to the left. **(A)** *MaCdx* is expressed in trunk discs of trunk-discs-stage larvae. A single trunk disc (td), cephalic disc (cd), and the stomach (st) are labeled for reference. **(B)** *MaCdx* expression in head-and-trunk stage occurs in posterior domains of each trunk rudiment (tr), with no evident expression in the dorsal disc (dd). The head rudiment (hr) is indicated for reference. **(C)** *MaCdx* is expressed just anterior to the juvenile caudal cirrus in extended-proboscis-stage larvae. **(A’-C’)** diagrammatically illustrate expression patterns in the three respective developmental stages.

In addition to the expression of *Hox* genes, we also examined the expression of *MaCdx* during pilidial development. *MaCdx* is first expressed in the trunk-discs stage in the posterior portion of trunk discs. By the head-and-trunk stage, *MaCdx* is expressed in the posterior portion of the trunk rudiments (Figure 6B). At the extended-proboscis stage, *MaCdx* is expressed in a ring around the juvenile trunk just anterior to the juvenile caudal cirrus (Figure 6C).

## Discussion

The trochozoan *Hox* complement typically contains 10 to 11 *Hox* genes [24-26]. Orthologs of all but two of the genes in the typical trochozoan repertoire were identified in *M. alaskensis*; we have not found orthologs of the central gene *Lox2* or of the posterior gene *Post1* in the *M. alaskensis* developmental transcriptome. Attempts to isolate *Lox2* and *Post1* from genomic DNA of *M. alaskensis* using degenerate PCR were not successful. Both genes have been found in annelids, mollusks, and brachiopods, but not in bryozoans [27]. *Post1* and *Post2* are likely a result of a gene duplication that occurred in the trochozoan lineage [21]. *Lox4* and *Lox2* are also related by a duplication event that likely took place within the Trochozoa [21]. One possible explanation for the absence of *Post1* and *Lox2* in *M. alaskensis* (if, indeed, the absence is real, and not a result of lack of expression, or low level of expression at surveyed developmental stages) is that the duplication events happened after the split between the Nemertea and the other trochozoan phyla. Alternatively, *Post1* and *Lox2* may have been lost in the nemertean lineage. *Post1* is not involved in axial patterning in either of the three annelid species [26,28,29] or in the two mollusk species in which posterior *Hox* genes have been analyzed. *Post1* is genomically disconnected from the rest of the cluster in the annelids *Capitella teleta* and *Platynereis dumerilii* [26,30]. This suggests that *Post1* may have been removed from participation in vectorial patterning in the trochozoan lineage. Perhaps, its absence in the *M. alaskensis* developmental transcriptome is related to an evolutionary loss of body patterning function.

The principal and remarkable finding here is that, based on their expression, all nine *Hox* genes in *M. alaskensis* likely participate in vectorial regionalization along the AP axis of the developing juvenile, but are not expressed at detectable levels in the pilidial larval body. Furthermore, it appears that *Hox* genes do not pattern the entire juvenile body, but only the posterior region, which develops from the trunk imaginal discs and dorsal rudiment. The canonical function of *Hox* genes in other bilaterians, such as mollusks [31-33], polychaetes [26,28,34-38], deuterostomes [39-41], and acoels [42,43], is AP patterning of the juvenile/adult trunk. Thus, with the caveat that we only have data for a single species, the role of *Hox* genes in axial patterning of the adult body appears to be conserved in nemerteans.

Although we do not have evidence from double labeling, based on the position of expression domains with respect to the morphological landmarks (for example, cerebral organ discs or the caudal cirrus), it is quite clear that these domains partially overlap along the AP axis of the juvenile body. Interestingly, the orthologs of anterior genes show earlier and more anterior patterns of expression compared to the orthologs of the more posterior genes. Because we do not know the genomic organization of *Hox* cluster in nemerteans (or even whether the genes are arranged in a cluster), it is unclear whether the spatial and temporal pattern of expression of these genes in pilidial development corresponds to the order of arrangement in the genome. Therefore we do not claim that *Hox* genes in *M. alaskensis* exhibit co-linearity. Nevertheless, it is noteworthy that the pattern of expression of *Hox* genes in *M. alaskensis* development corresponds to the order of arrangement of their orthologs in animals with known genomic organization of the *Hox* cluster (for example, *C. teleta* [26], *Lottia gigantea* [25], and *Branchiostoma floridae* [44]).

One of the *Hox* genes, *MaScr*, has a peculiar pattern of expression: in a few small patches on the lateral surface of the juvenile trunk just anterior to its belt-like domain. The position of these dots roughly corresponds to the position of the nephridial openings in the juvenile trunk of *M. alaskensis* [19]. We do not claim here that this is what they are, because we do not have an easy way to label nephridial openings in the same preparation as the *in situ* hybridization, but the pattern is suggestive.

The *ParaHox* gene *Cdx* patterns posterior structures in a number of spiralians, such as the gastropods *Gibbula varia* and *Patella vulgate*, the polychaete *Platynereis dumerilii,* and the nemertean *Lineus viridis* [15,45]. Similarly, in *M. alaskensis* it is expressed in the posterior end of the developing juvenile.

Because *Hox* genes are not expressed during *M. alaskensis* embryogenesis or at any other time in the larval tissues, the pilidial body must be patterned by mechanisms other than *Hox* genes. At this time it is unclear whether the pilidium larva and juvenile share other patterning mechanisms, or if the pilidial AP axis is patterned by a novel mechanism. Preliminary experiments with inhibitors of the Wnt and fibroblast growth factor pathways suggest that these may be involved in patterning the pilidium (Hiebert, unpublished).

*Hox* gene expression has been studied in many other animals, but most of those develop more or less directly [41,46,47]. In most direct-developers, the adult axial properties can be traced directly back to initial asymmetries in the egg [48,49]. Even in some indirect-developers, such as some annelids or the fruit fly, a blueprint of the adult is already present at the larval stage [28,49,50]. In the pilidium, there is no clear early blueprint of the adult worm, and we found that *Hox* patterning is not shared between early pilidial and adult stages. The same is true for another group of maximally indirectly developing animals: the sea urchins. In urchins, the *Hox* genes are also more or less limited to patterning the juvenile structures as they form inside the larva [51,52]. But, in urchins, the adult body is highly modified and the *Hox* expression pattern is difficult to relate to that in other bilaterians. Nemertean adult body, however, is clearly comparable to that of other bilaterians. It is remarkable that *Hox* genes maintain conserved expression in the juvenile body of *M. alaskensis* even though it develops separately from the larva and expression of *Hox* genes is evident even before the individual juvenile rudiments form a contiguous domain.

Expression patterns of *Hox* and *Cdx* genes in pilidial development suggest that the larval body may represent an ontogenetic ‘insertion’ into a more direct developmental program because these adult patterning mechanisms are not shared by the larva. Thus, less constraint may exist on phase-specific evolution in the pilidiophorans than in animals whose larval stage preforms the adult. This may be relevant to how the pilidium arose, diversified, and how catastrophic metamorphosis evolved in nemerteans.

## Conclusions

We find that *Hox* genes in the pilidiophoran *Micrura alaskensis* pattern the AP axis of the juvenile trunk as it arises from isolated rudiments inside the pilidium larva. The pilidium, an evolutionarily novel body plan, is patterned without the use of *Hox* genes. The lack of shared axial patterning mechanisms (*Hox* and *Cdx*) across the two life stages may help explain the extreme morphogenetic uncoupling between the larva and the juvenile.

## Methods

### Collection of adults, fertilization of gametes, and larval culture

Adult *M. alaskensis* Coe, 1901 (Heteronemertea; Lineidae) were collected from mudflats in Coos Bay near Charleston, OR, USA during May to August of 2009 to 2013 on negative tides. Adult worms were transported to the Oregon Institute of Marine Biology and kept in a flow-through seawater system in 150 mL glass custard dishes until dissection. Adults were either collected bearing ripe gametes or allowed to ripen in the laboratory over a few weeks. Primary oocytes and sperm were dissected from live females and males and transferred to 0.45 μm filtered seawater (FSW). After around 30 minutes, oocytes underwent germinal vesicle break down and were fertilized with a dilute suspension of sperm. Larval culture followed methods described by Maslakova [19]. In short, embryos were kept in custard dishes until reaching a swimming stage, at which point the larvae were transferred to gallon jars kept at ambient sea temperature (12 to 16°C) with constant stirring using acrylic paddles [53]. Larval concentrations during the first few weeks of development were approximately one larval per milliliter. Subsequently, larvae were maintained at close to one larva per 10 mL. FSW was exchanged every 2 to 3 days via reverse-filtration. The larvae were fed *Rhodomonas lens* (10^4^ cells/mL) after each water change.

### Isolation of *Hox* genes

*Hox-*containing contigs were retrieved from a *M. alaskensis* developmental transcriptome containing transcripts from seven developmental stages (gastrula, young feeding pilidium, cephalic-discs stage, cerebral-organ-discs stage, head-and-trunk stage, and hood to pre-metamorphosis stages). The assembly contained nine unique *Hox* gene transcripts, seven of which included a full open reading frame. Long fragments of each *Hox* gene were amplified from cDNA libraries from torus stage and extended-proboscis- to hood-stage larvae. Primers were designed from the transcriptome contigs using Primer3 [54,55]. Additional sequence fragments were amplified by rapid amplification of cDNA ends (RACE [56]) from cDNA libraries derived from head-and-trunk-stage, torus-stage, and extended-proboscis-stage to hood-stage larvae. PCR and RACE products were subcloned into PGEM-t (Promega, Madison, WI, USA) vectors and then transformed into One Shot Top10 chemically competent *Escherichia coli* cells (Invitrogen, Carlsbad, CA, USA). Plasmid DNA was isolated using QIAprep Spin Miniprep Kit (Qiagen Valencia, CA, USA) and sequenced in both forward and reverse directions on a ABI 3730xl DNA Analyzer platform (Sequetech, Mountain View, CA, USA) using T7 and SP6 primers.

### Orthology assessment and phylogenetic analysis

Orthology was determined by the presence of characteristic residues in the homeodomain and flanking regions and by phylogenetic analysis. An amino acid sequence alignment was made by eye using Geneious version 7.0.4 [57] and included the complete homeodomain, the 12 amino acids just 3′ of the homeodomain, and the eight amino acids just 5′ of the homeodomain. *Hox* and *Cdx* fragments from *M. alaskensis* were aligned with *Cdx* and *Hox* complements of a deuterostome *Branchiostoma florida*, two ecdysozoans (*Tribolium castaneum*, *Drosophila melanogaster*), and five lophotrochozoans (an annelid *C. teleta*, a bryozoan *Bugula turrita*, a nemertean *Ramphogordius (=Lineus) sanguineus*, a brachiopod *Lingula anatina*, and a mollusk *Euprymna scolopes*). Sequence data from representative metazoans were retrieved using the National Center for Biotechnology ([58]; accession numbers listed in Additional file 5).

Phylogenetic analysis (Bayesian inference) was conducted using MrBayes version 3.2.1 [59,60]. Hox fragments were aligned using the homeodomain and the twelve 3′ amino acids. The analysis was done with the Rtrev amino acid model with a gamma-shaped distribution of rates across sites. *Drosophila eve* was selected as an out-group. The analysis was done with five heated chains with 5,000,000 generations and was sampled every 500 generations. Four independent runs were conducted. The first 25% samples from the cold chain was discarded as burn-in. Trees were visualized and manipulated using Figtree version 1.4.0.

### Whole mount *in situ* hybridization

Gastrulae and larvae were relaxed in 1:1 mixture of 0.37 M MgCl_2_ in FSW for 10 minutes, then fixed in 4% paraformaldehyde in FSW overnight at 4°C. Fixed larvae were washed three times in 1X phosphate-buffered saline (PBS), dehydrated in methanol, and stored at −20°C. A protocol for *in situ* hybridization was modified from Seaver and Kaneshige [61]. Larvae were rehydrated in PBS, followed by three washes in PBS with 0.1% Tween-20 (PTw). All wash and incubation steps were 5 to 10 minutes, unless otherwise indicated. Fixed tissues were acetylated in 1% triethanolamine in PTw with 0.3% acetic anhydride, and then incubated in 1% triethanolamine in PTw with 0.6% acetic anhydride. After two quick rinses with PTw, larvae were re-fixed with 4% paraformaldehyde for 30 to 60 minutes. The fixative was removed with four washes of PTw. Larvae were transferred to hybridization buffer (50% formamide, 5X saline-sodium citrate (SSC)pH7, 50 μg/mL heparin, 0.1% Tween-20, 1% SDS, 50 μg/mL boiled salmon sperm DNA made up in diethyl dicarbonatewater) at hybridization temperature (63°C) for 4 hours to overnight. Digoxygenin-labeled riboprobes were synthesized using linearized template DNA using DIG RNA Labeling Mix and following standard protocol (Roche, Penzberg, Germany). Probes were quantified using a Qubit fluorometer (Invitrogen) then diluted to 1 ng/μL in hybridization buffer and either used right away or stored at −20°C. Diluted probes were saved after hybridization and reused up to five times. Before hybridization, probes were denatured at 80°C to 90°C for 10 minutes. Larvae were hybridized with the probe in hybridization buffer at 63°C for 2 to 3 days. After hybridization, the probe was washed out with hybridization buffer. Then larvae were washed through a graded series of hybridization buffer/2X SSC (75/25, 50/50, 25/75, 100/0). Larvae were washed of excess probe by incubating in 0.05X SSC for 30 minutes at 63°C. This step was repeated once, followed by transfer through a series (70/30, 30/70, 0/100) to Tris-buffered saline and Tween (TBST; 0.15 M NaCl; 0.2 M Tris buffer, pH 7.5). Larvae were washed four times in TBST, then blocked in TBST with 0.1% Tween-20, 5% normal goat serum, and 2 ng/μL bovine serum albumin). The color reaction took place in 1X Detection Buffer (Roche) with 4.4 μL of 75 mg/mL Nitro Blue Tetrazolium (Sigma, St. Louis, MO, USA) and 3.3 μL of 50 mg/mL 5-bromo-4-chloro-3-indolyl phosphate (Sigma). Staining was carried out in the dark from 1 hour to 2 days, depending on the probe. The staining reaction was terminated by washing larvae in PTw. Stained larvae were mounted on slides in 80% glycerol in PBS. Photomicrographs were obtained using a Leica DFC400 digital color camera mounted on an Olympus BX51 microscope equipped with differential interference contrast optics. Ten to twenty specimens were examined for each gene and stage.

## Additional files

Additional file 1:
**Alignment of** ***Hox*** **and** ***Cdx*** **genes and flanking sequences.** Alignment includes the *Hox* homeodomain, twelve residues 3′ of the homeodomain, and eight residues 5′ of the homeodomain for representative species. Sequences are aligned against *Drosophila melanogaster Antp*. Yellow highlighting indicates conserved residues that differ from other paralog groups. Green highlighting indicates positions of conserved peptide motifs outside the homeodomain. Additional file 2:
**Bayesian phylogenetic analysis of** ***M. alaskensis Hox*** **genes.** Bf, *Branchiostoma floridae*; Tc, *Tribolium castaneum*; Dm, *Drosophila melanogaster*; Ct, *Capitella teleta*; Bt, *Bugula turrita*; Ls, *Lineus sanguineus*; La, *Lingula anatine*; Es, *Euprymna scolopes*. Numbers at branch points indicate Bayesian posterior probabilities. Values lower than 50% are not included. Colors denote paralog groups (PG). Arrowheads indicate location of *M. alaskensis Hox* genes. Additional file 3:
**Table of** ***Hox, Cdx,*** **and** ***Six3/6*** **genes isolated from** ***M. alaskensis*** **.**
Additional file 4:
***MaSix3/6*** **expression in** ***M. alaskensis*** **development.** **(A)** Polar view (animal or vegetal) of blastosquare. **(B-D)** Feeding pilidium, lateral view. Stomach (st), cephalic disc (cd), and trunk disc (td) labeled. Asterisk marks apical organ. (A’-D’) diagrammatically illustrate expression patterns in the respective developmental stages. Additional file 5:
**Table of sequences used for alignment and phylogenetic analysis.**
