# Supplementary material for: Hox genes pattern the anterior-posterior axis of the juvenile but not the larva in a maximally indirect developing invertebrate, Micrura alaskensis (Nemertea)
Source: BMC Biol. 2015 Apr 11;13:23. doi: 10.1186/s12915-015-0133-5 (PMC4426647; doi:10.1186/s12915-015-0133-5)
Supplement: Additional file 1: — Alignment of Hox and Cdx genes and flanking sequences. Alignment includes the Hox homeodomain, twelve residues 3′ of the homeodomain, and eight residues 5′ of the homeodomain for representative species. Sequences are aligned against Drosophila melanogaster Antp. Yellow highlighting indicates conserved residues that differ from other paralog groups. Green highlighting indicates positions of conserved peptide motifs outside the homeodomain. [file 12915_2015_133_MOESM1_ESM.pdf]

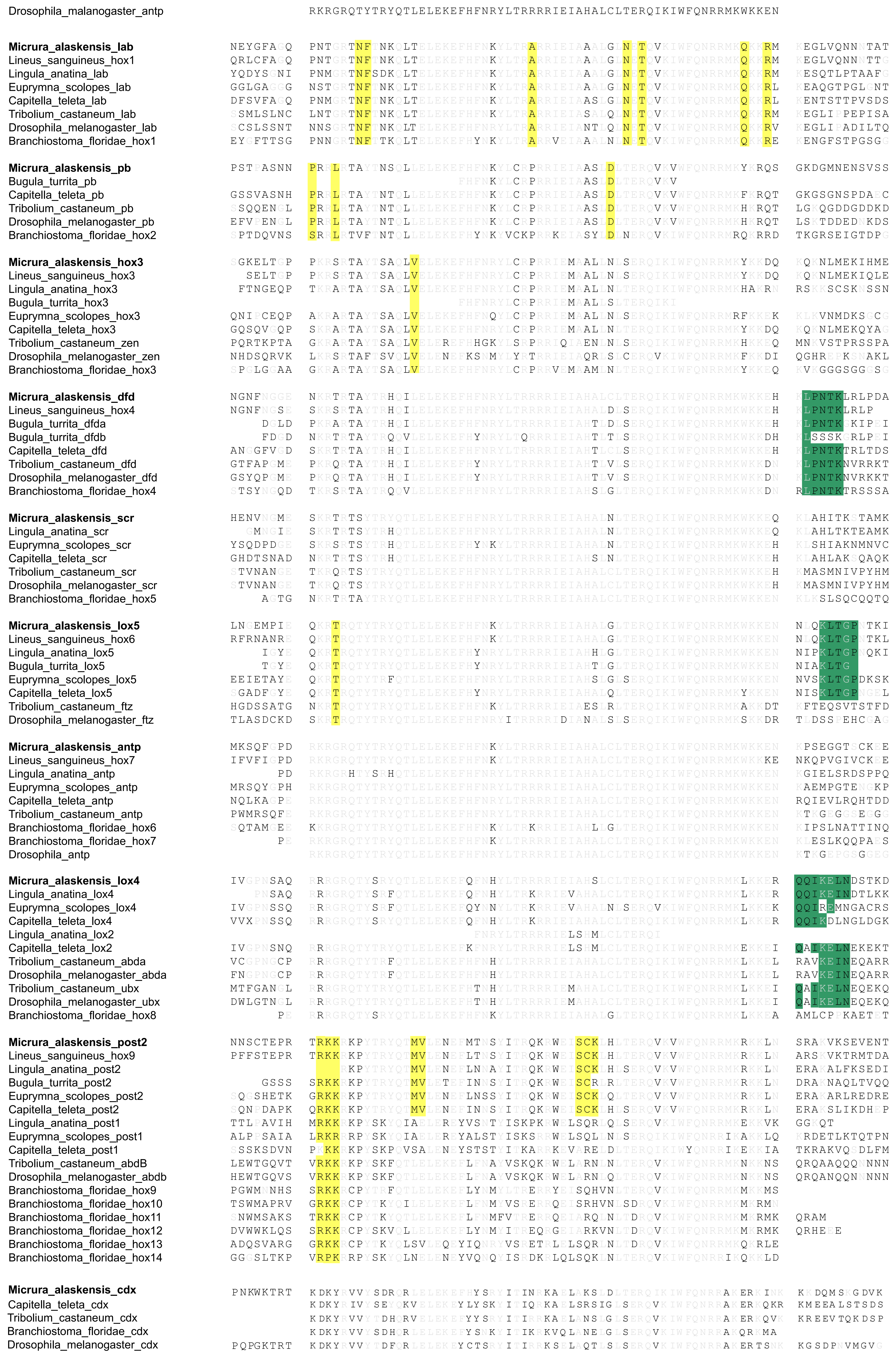

Drosophila_malanogaster_antp
RKRGRQTYTRYQTLELEKEFHFNRYLTRRRRIEIAHALCLTERQIKIWFQNRRMKWKKEN
Micrura_alaskensis_lab
Lineus_sanguineus_hox1
Lingula_anatina_lab
Euprymna_scolopes_lab
Capitella_teleta_lab
Tribolium_castaneum_lab
Drosophila_melanogaster_lab
Branchiostoma_floridae_hox1
Micrura_alaskensis_pb
Bugula_turrita_pb
Capitella_teleta_pb
Tribolium_castaneum_pb
Drosophila_melanogaster_pb
Branchiostoma_floridae_hox2
Micrura_alaskensis_hox3
Lineus_sanguineus_hox3
Lingula_anatina_hox3
Bugula_turrita_hox3
Euprymna_scolopes_hox3
Capitella_teleta_hox3
Tribolium_castaneum_zen
Drosophila_melanogaster_zen
Branchiostoma_floridae_hox3
Micrura_alaskensis_dfd
Lineus_sanguineus_hox4
Bugula_turrita_dfda
Bugula_turrita_dfdb
Capitella_teleta_dfd
Tribolium_castaneum_dfd
Drosophila_melanogaster_dfd
Branchiostoma_floridae_hox4
Micrura_alaskensis_scr
Lingula_anatina_scr
Euprymna_scolopes_scr
Capitella_teleta_scr
Tribolium_castaneum_scr
Drosophila_melanogaster_scr
Branchiostoma_floridae_hox5
Micrura_alaskensis_lox5
Lineus_sanguineus_hox6
Lingula_anatina_lox5
Bugula_turrita_lox5
Euprymna_scolopes_lox5
Capitella_teleta_lox5
Tribolium_castaneum_ftz
Drosophila_melanogaster_ftz
Micrura_alaskensis_antp
Lineus_sanguineus_hox7
Lingula_anatina_antp
Euprymna_scolopes_antp
Capitella_teleta_antp
Tribolium_castaneum_antp
Branchiostoma_floridae_hox6
Branchiostoma_floridae_hox7
Drosophila_antp
Micrura_alaskensis_lox4
Lingula_anatina_lox4
Euprymna_scolopes_lox4
Capitella_teleta_lox4
Lingula_anatina_lox2
Capitella_teleta_lox2
Tribolium_castaneum_abda
Drosophila_melanogaster_abda
Tribolium_castaneum_ubx
Drosophila_melanogaster_ubx
Branchiostoma_floridae_hox8
Micrura_alaskensis_post2
Lineus_sanguineus_hox9
Lingula_anatina_post2
Bugula_turrita_post2
Euprymna_scolopes_post2
Capitella_teleta_post2
Lingula_anatina_post1
Euprymna_scolopes_post1
Capitella_teleta_post1
Tribolium_castaneum_abdB
Drosophila_melanogaster_abdb
Branchiostoma_floridae_hox9
Branchiostoma_floridae_hox10
Branchiostoma_floridae_hox11
Branchiostoma_floridae_hox12
Branchiostoma_floridae_hox13
Branchiostoma_floridae_hox14
Micrura_alaskensis_cdx
Capitella_teleta_cdx
Tribolium_castaneum_cdx
Branchiostoma_floridae_cdx
Drosophila_melanogaster_cdx
