# Supplementary figures and images for: Hox genes pattern the anterior-posterior axis of the juvenile but not the larva in a maximally indirect developing invertebrate, Micrura alaskensis (Nemertea)
Source: BMC Biol. 2015 Apr 11;13:23. doi: 10.1186/s12915-015-0133-5 (PMC4426647; doi:10.1186/s12915-015-0133-5)

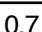

Supplement: Additional file 2: — Bayesian phylogenetic analysis of M. alaskensis Hox genes. Bf, Branchiostoma floridae; Tc, Tribolium castaneum; Dm, Drosophila melanogaster; Ct, Capitella teleta; Bt, Bugula turrita; Ls, Lineus sanguineus; La, Lingula anatine; Es, Euprymna scolopes. Numbers at branch points indicate Bayesian posterior probabilities. Values lower than 50% are not included. Colors denote paralog groups (PG). Arrowheads indicate location of M. alaskensis Hox genes. [file 12915_2015_133_MOESM2_ESM.pdf]

A

*MaSix3/6*50  $\mu\text{m}$ 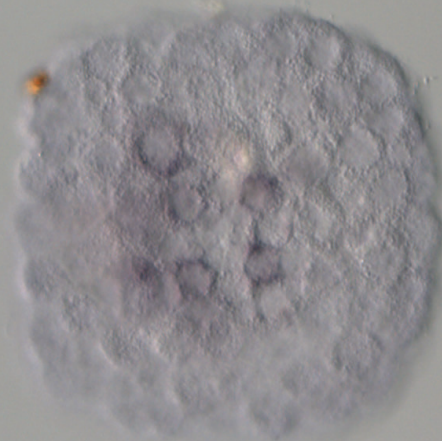

A'

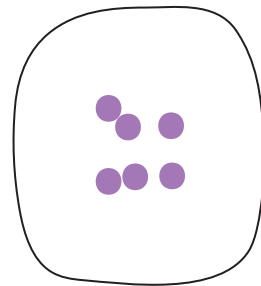

B

*MaSix3/6*  
\*40  $\mu\text{m}$ 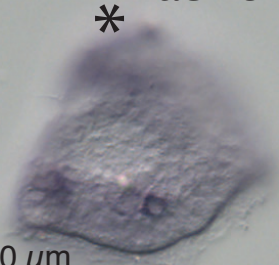

C

*MaSix3/6*  
\* st40  $\mu\text{m}$ 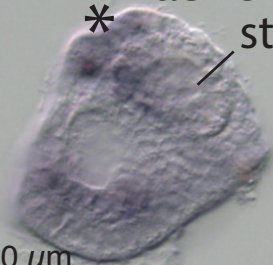

D

*MaSix3/6*  
\*40  $\mu\text{m}$ 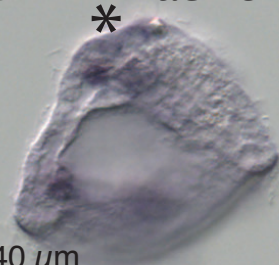

BCD'

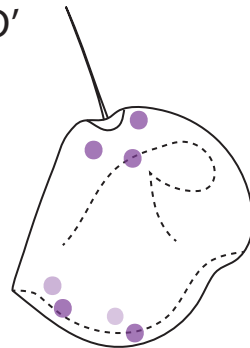

Supplement: Additional file 4: — MaSix3/6 expression in M. alaskensis development. (A) Polar view (animal or vegetal) of blastosquare. (B-D) Feeding pilidium, lateral view. Stomach (st), cephalic disc (cd), and trunk disc (td) labeled. Asterisk marks apical organ. (A’-D’) diagrammatically illustrate expression patterns in the respective developmental stages. [file 12915_2015_133_MOESM4_ESM.pdf]
