# Supplementary material for: Hox genes pattern the anterior-posterior axis of the juvenile but not the larva in a maximally indirect developing invertebrate, Micrura alaskensis (Nemertea)
Source: BMC Biol. 2015 Apr 11;13:23. doi: 10.1186/s12915-015-0133-5 (PMC4426647; doi:10.1186/s12915-015-0133-5)
Supplement: Additional file 3: — Table of Hox, Cdx, and Six3/6 genes isolated from M. alaskensis . [file 12915_2015_133_MOESM3_ESM.doc]

Additional file 3

| Paralog group | Gene name | Probe length | Predicted ORF | Genbank accession # |
| --- | --- | --- | --- | --- |
| PG1 | *Lab* | 1072 bp | 831 bp | KP762174 |
| PG2 | *Pb* | 707 bp | missing 3’ end | KP762176 |
| PG3 | *Hox3* | 1129 bp | 1125 bp | KP762173 |
| PG4 | *Dfd* | 1078 bp | 810 bp | KP762180 |
| PG5 | *Scr* | 1149 bp | 474 bp | KP762177 |
| PG6 | *Lox5* | 1069 bp | 720 bp | KP762179 |
| PG7 | *Antp* | 1023 bp | 708 bp | KP762171 |
| PG8 | *Lox4* | 746 bp | missing 5’ end | KP762175 |
| PG9-15 | *Post2* | 962 bp | 738 bp | KP762178 |
| N/A | *Cdx* | 943 bp | 1082 bp | KP762170 |
| N/A | *Six3/6* | 756 bp | 573 bp | KP762172 |
