# Supplementary material for: Hox genes pattern the anterior-posterior axis of the juvenile but not the larva in a maximally indirect developing invertebrate, Micrura alaskensis (Nemertea)
Source: BMC Biol. 2015 Apr 11;13:23. doi: 10.1186/s12915-015-0133-5 (PMC4426647; doi:10.1186/s12915-015-0133-5)
Supplement: Additional file 5: — Table of sequences used for alignment and phylogenetic analysis. [file 12915_2015_133_MOESM5_ESM.doc]

Additional file 5

| Species | Gene names | Accession # |
| --- | --- | --- |
| Branchiostoma floridae | Bf_hox1 Bf_hox2  Bf_hox3  Bf_hox4  Bf_hox5 Bf_hox6 Bf_hox7 Bf_hox8 Bf_hox9 Bf_hox10 Bf_hox11 Bf_hox12 Bf_hox13 Bf_hox14  Bf_cdx | BAA78620 BAA78621  X68045  BAA78622 CAA84517 CAA84518 CAA84519 CAA84520 CAA84521 CAA84522 AAF81909 AAF81903 AAF81904 AAF81905  AAC39017 |
| Bugula turrita | Bt_pb Bt_hox3 Bt_dfda Bt_dfdb  Bt_lox5  Bt_post2 | AAS77225  AAS77226  AAS77227  AAS77228  AAS77229  AAS77230 |
| Capitella teleta | Ct_lab  Ct_pb  Ct_hox3  Ct_dfd  Ct_scr  Ct_lox5  Ct_antp  Ct_lox4  Ct_lox2  Ct_post1  Ct_post2  Ct_eve  Ct_cdx | ABY67952  ABY67953  ABY67954  ABY67955  ABY67956  ABY67957  ABY67962  ABY67958  ABY67959  ABY67961  ABY67960  ABG82164  AAZ95508 |
| Drosophila melanogaster | Dm_lab Dm_pb  Dm_zen  Dm_dfd Dm_scr Dm_ftz Dm_antp Dm_ubx Dm_abdA Dm_abdB  Dm_eve  Dm_cdx | CAB57787  CAA45271  P09089  P07548  NP_524248  NP_477498  CAA27417  CAA29194  P29555  CAB57859  AAF58865  AAA28409 |
| Euprymna scolopes | Es_lab Es_hox3 Es_scr Es_lox5 Es_antp Es_lox4 Es_post1 Es_post2  Es_cdx | AY330184  AY330185  AY330186  AY330187  AY330188  AY330189  AY330190  AY330191  AF127341 |
| Lingula anatina | La_lab La_hox3 La_scr La_lox5 La_antp La_lox2 La_lox4 La_post2 La_post1 | AAD45587  AAD45588  AAD45589  AAD45591  AAD45590  AAD45592  AAD45593  AAD45595  AAD45594 |
| Lineus sanguineus | Ls_hox1 Ls_hox3  Ls_hox4  Ls_hox6  Ls_hox7  Ls_hox9 | CAA76295 CAA76296  P81192  CAA76297 CAA76298 CAA76299 |
| Tribolium castaneum | Tc_lab  Tc_pb  Tc_zen  Tc_dfd  Tc_scr  Tc_ftz  Tc_antp  Tc_ubx Tc_abdA Tc_abdB  Tc_eve  Tc_cdx | NP_001107762  EEZ99256  NP_001036813  NP_001034510  AAK16422 NP_001034539  EEZ99250  NP_001034497 NP_001034518 NP_001034519  NP_001034538  NP_001034498 |
